# Long-term effects of a 12 weeks high-intensity functional exercise program on physical function and mental health in nursing home residents with dementia: a single blinded randomized controlled trial

**DOI:** 10.1186/s12877-015-0151-8

**Published:** 2015-12-03

**Authors:** Elisabeth Wiken Telenius, Knut Engedal, Astrid Bergland

**Affiliations:** Department of physiotherapy, Faculty of Health Sciences, Oslo and Akershus University College of Applied Sciences, PO Box 4 St Olavs plass, 0130 Oslo, Norway; Norwegian Centre of Aging and Health, Department of Psychiatry, Vestfold Health Trust, Tønsberg and Oslo University Hospital, Oslo, Norway

**Keywords:** Exercise, Dementia, Nursing home, Aged, Balance, Activities of daily living, Neuropsychiatric symptoms, Agitation

## Abstract

**Background:**

Research indicates that exercise can have a positive effect on both physical and mental health in nursing home patients with dementia, however the lasting effect is rarely studied. In a previously published article we investigated the immediate effect of a 12 weeks functional exercise program on physical function and mental health in nursing home residents with dementia. In this paper we studied the long-term effect of this exercise program. We explored the differences between the exercise and control group from baseline to 6 months follow-up and during the detraining period from month 3 to 6.

**Methods:**

A single blind, randomized controlled trial was conducted and a total of 170 nursing home residents with dementia were included. The participants were randomly allocated to an intervention (*n* = 87) or a control group (*n* = 83). The intervention consisted of intensive strengthening and balance exercises in small groups twice a week for 12 weeks. The control condition was leisure activities. Thirty participants were lost between baseline and six-month follow-up. Linear mixed model analyses for repeated measurements were used to investigate the effect of exercise after detraining period.

**Results:**

The exercise group improved their scores on Berg Balance Scale from baseline to 6 months follow-up by 2.7 points in average. The control group deteriorated in the same period and the difference between groups was statistically significant (*p* = 0.031). The exercise group also scored better on NPI agitation sub-score after 6 months (*p* = 0.045).

**Conclusion:**

The results demonstrate long-time positive effects of a high intensity functional exercise program on balance and indicate a positive effect on agitation, after an intervention period of 12 weeks followed by a detraining period of 12 weeks.

Identifier at ClinicalTrials.gov: NCT02262104

## Background

The world population is rapidly aging and as a result, health and social care services will come under pressure to provide services for older people with dementia as well as persons with a wider range of other chronic diseases. Dementia is among the leading causes of disability and death in the elderly [1, 2]. There is no cure for dementia [3] and the on-going degeneration of brain tissue in older adults with neurodegenerative dementia disorders eventually leads to a loss of cognitive and physical functions [4, 5]. About 80 % of nursing home residents in Norway suffer from dementia [6], and it is the most common main diagnosis in the nursing home population in Norway [7].

Individuals with dementia have higher levels of functional dependence than others and are therefore more likely to require assistance in activities of daily living (ADL) [8, 9]. In addition to impaired cognition, reduced ADL function and changed behaviour, dementia normally affects balance, mobility, and gait performance [10–15]. Reduced balance increases the risk of falling, and falls and fractures are common among nursing home residents with dementia [10, 11, 16, 17]. People with dementia have a two-fold increased risk of falls compared with non-demented elderly [16]. Fear of falling itself is a risk factor for inactivity and can create a vicious circle [18]. Therefore, improvements in balance may potentially reduce the risk of falling and increase mobility through increased confidence.

As dementia progresses, cognitive and functional impairments are followed by neuropsychiatric symptoms (NPS) [19]. Studies show that more than 80 % of persons with dementia in nursing homes have at least one clinically significant NPS [20, 21] and behavioural symptoms are one of the main reasons for institutionalization [22]. The most prevalent symptoms in patients with dementia in nursing homes are agitation, apathy and affective symptoms [20, 21, 23]. These symptoms cause discomfort and reduced quality of life for people with dementia and they are predictors of fall for nursing home patients, causing considerable morbidity and mortality [24, 25]. NPS also give distress to family and carers .

The effect of exercise on mental health is well established in other groups, however in the population of nursing home residents with dementia, most studies focus on the impact of exercise on physical functioning and mobility, rather than neuropsychiatric symptoms and cognition. Therefore research on the effect of exercise on agitation in dementia is scarce. In a systematic review of non-pharmacological interventions for agitation in dementia from 2009, no exercise interventions were included due to low methodological quality [26]. Historically, these symptoms have been managed with anxiolytic and antipsychotic medications [27]. Although potentially effective, such medication has been used too widely and may be associated with serious adverse side effects and increased mortality [28]. According to Ballard et al., 2006, over prescribing has become a major problem, especially in residential and nursing home environments. It is reported that 42–66 % of people with dementia are taking antipsychotic drugs [6, 29–31], often inappropriately and usually with little monitoring [32]. Antipsychotic drugs have substantial adverse effects such as increased risk of falls [33, 34] and increased mortality [35]. Consequently, there is a need to evaluate non-pharmacological therapies for behavioural and neuropsychiatric symptoms in this population [36].

Reviews have concluded that there is insufficient evidence for the effectiveness of physical activity on function, cognition, neuropsychiatric symptoms and depression in older people with dementia [36–40]. Difficulties with measurements and instructions and lack of compliance have led the majority of studies on physical exercise to exclude people with dementia. In a previous study we demonstrated the effect of high intensity functional exercises on balance, strength and apathy in nursing home residents with mild and moderate dementia, as measured after a 12 weeks program [41]. Muscle strength gains induced by resistance training programs are lost after short detraining period [42–45] and there are reasons to believe that this will happen even more rapidly in nursing home residents due to the sedentary lifestyle [46]. The detraining effect on balance is less investigated, but it seems that the balance function as well declines abruptly after completion of an exercise program [47–49]. There are several reasons as to why it is important to investigate the detraining period. First of all, elderly people are more prone to interrupt exercise program due to ill health, and information about what happens to the exercise effects and when, is important to optimize quality of care. Research studies often have a rather short intervention period, e.g. 12 weeks and some have shown that the immediate effect of exercise can be different from the effect weeks later [50]. It has been suggested that different types of exercise, intensity and age may also influence detraining effects [43, 45, 51] and this needs to be investigated. From an economic point of view, it can be interesting to find out how little you can “get away” with.

As demonstrated above, randomized, controlled trials with physical exercise among nursing home residents with dementia are limited and knowledge about long-term effects are lacking. In a previously published article we investigated the immediate effect of a 12 weeks exercise program on physical function and mental health in nursing home residents with dementia [41]. An effect of exercise on balance, the primary outcome, was demonstrated. In this paper we would like to study the long-term effect of this exercise program. Is there still a significant difference in balance function between the two groups 3 months after exercise cessation? We aimed to investigate the change in physical function and mental health from baseline to 6 months follow-up and during the detraining period, in the two groups.

## Methods

### Design

Exercise and dementia – EXDEM – was a 12-week single blinded parallel multi centre randomized controlled trial, followed by a 12 weeks detraining period, conducted among nursing home residents with dementia. Participants in 18 different nursing homes were randomly allocated to two groups: physical exercise and control activity. We used a block randomization procedure with six to 12 participants from each nursing home. Due to great heterogeneity between the nursing homes regarding residents’ demographics, care staff awareness concerning importance of physical activity and physical activity-opportunities, we considered it to be most appropriate to use block randomization.

Identifier at ClinicalTrial.gov: NCT02262104, registered November 2013.

### Participants

The study took part in 18 nursing homes in Oslo city areas in Norway between May 2012 and September 2013. Each nursing home was given the opportunity to recruit up to 12 participants each, which gives a total of 216 possible participants. Inclusion criteria were: being above 55 years of age, having dementia of mild or moderate degree as measured by the Clinical Dementia Rating scale (CDR 1 or 2), being competent to consent to participation, being able to stand up alone or by the help of one person and being able to walk six meters with or without walking aid. Exclusion criteria: patients being medically unstable, psychotic or having severe communication problems.

**Fig. 1:**
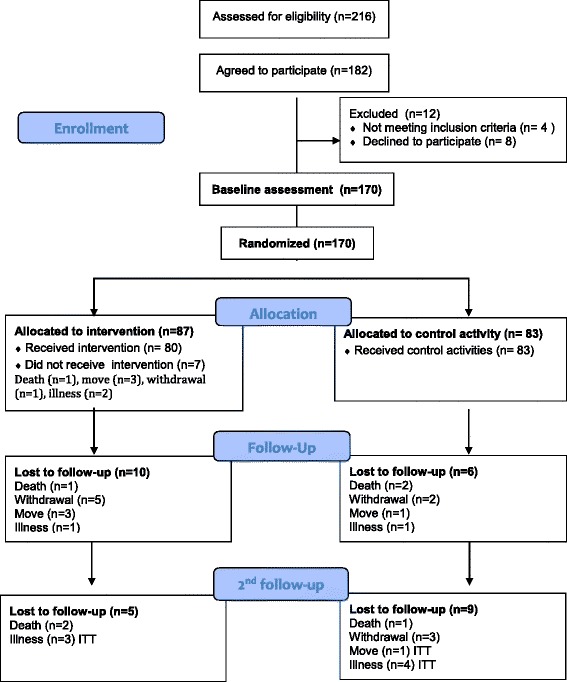
Flowchart of the study populationᅟ

In total, 182 persons (84 %) agreed to participate. Twelve persons (6.6 %) dropped out of the study before the first assessment and randomization was carried out: eight withdrew and four were excluded because the inclusion criteria were not met (See flowchart Fig. 1). Thus, the study population consisted of 170 participants. A further 16 participants were lost to follow up at 12 weeks (3 died, 7 withdrew, 4 moved to another nursing home and 2 became seriously ill). At 24 weeks follow-up a further 14 were lost (3 died, 3 withdrew, 1 moved and 7 became seriously ill). See Flowchart, Fig. 1. The participants who were lost to follow-up were excluded from the analysis.

### Randomization

An independent trial secretary performed the randomization procedure. Following completion of the pre-intervention assessments the participants from each nursing home were randomly assigned to either intervention or control group by picking name from a container. The randomization codes were kept in sealed envelopes with consecutive numbering by the trial secretary and blinded to those who assessed the patients.

### Physical exercise program

Three to six participants at each nursing home exercised twice a week for 12 weeks with physiotherapists (1 physiotherapist per 3 participants). The exercise program was the High Intensity Functional Exercises (HIFE) –program developed in Umeå, Sweden [52]. The exercise sessions lasted 50–60 min and consisted of: 5 min warm-up, at least two strengthening exercises for the muscles of lower limb and two balance exercises. All exercises were individually tailored, instructed and supervised. The intensity of strengthening exercises aimed to be 12 repetitions maximum (RM). The balance exercises intended to be “highly challenging”. The physiotherapists kept detailed records of all exercise sessions and reported the type and number of exercises performed, the intensity (low, moderate or high) and any adverse occurrences. For more information about HIFE and the exercise sessions, see [52] Local physiotherapists (i.e. employed at the nursing home) were used wherever possible. Nine nursing homes (50 %) received help from one (*n* = 7) or two (*n* = 2) external physiotherapists to be able to participate in the study. In total, 27 physiotherapists were involved in the intervention program and all of them had attended a course to learn about the HIFE-program. The course lasted three hours and included practical exercises. The importance of targeting high intensity was emphasized. Adjustable weighted belts (0.5–12 k) were made available to all participating nursing homes. The project leader kept in touch with all physiotherapists during the intervention period through meetings (one obligatory), observation of exercise sessions (often in conjunction with the obligatory meeting), and by phone and email. This was done to optimize quality, high intensity and uniform execution in all nursing homes

The participants were not blinded after assignments to intervention.

### Control program

The control participants met in groups twice a week for 50–60 min and the activities were led by occupational therapists (*n* = 11), nursing staff (*n* = 5), volunteers (*n* = 1) or activity-leader (*n* = 1). The control activities were light physical activity in sitting (mostly mobility exercises and stretches), reading, playing games, listening to music and making conversations.

### Detraining

After the 12-week intervention (HIFE and control program) the participants received no further intervention or attention from any of the physical or occupational therapists. No specific advice about physical activity during the detraining period was given, except that there should be no difference between the participants of the two groups.

### Assessments

After enrolment, but before randomization, all 170 participants were assessed. A member of the nursing staff, who knew the participant well and was in regular contact with him/ her, filled in the Case Record Form. Nursing staff members that were not familiar with the questionnaires were encouraged to contact the project leader with any questions. An occupational therapist or specially trained nurse performed the Mini-Mental State Examination (MMSE). Four physiotherapists from the research team without knowledge of group allocation (blinded) performed all physical tests. Information about the testers training can be found in a previous paper [41]. The assessment procedure was repeated after 12 weeks (intervention completion) and 6 months and was carried out December 2012 to September 2013.

### Instruments

#### Physical function-tests

The primary outcome was *balance* measured by the Berg Balance Scale (BBS). The test assesses performance on a 5-level scale from 0 (cannot perform) to 4 (normal performance) on 14 different tasks involving functional balance control, including transfer, turning and stepping [53]. The six-meter walking test at comfortable speed with or without a walking aid tested *mobility* in the participants. The time in seconds was recorded and calculated as meters per second [54]. *Muscle strength* was measured by the 30-s chair stand test (CST). The score equals the number of rises from the chair in 30 s [55]. The physical tests used in this study were tested for inter-tester reliability in 33 of the participants. The tests were found to have high inter-tester reliability (ICC ≥ 0.97 on BBS, CST and NWS) [56]. To measure the patients’ dependence in the *ADL,* we employed the Barthel Index (BI), a widely used measure of the activities of daily living [57, 58]. The BI consists of 10 activities focusing on the patient’s level of dependence on aid. The scores range from 0 (completely dependent) to 20 (independent). Professional caregivers filled out the BI-questionnaire based on their observations of the participants.

### Dementia, cognition and neuropsychiatric symptoms

The Clinical Dementia Rating Scale (CDR), and the Mini-Mental State Examination (MMSE) were used to measure *cognition*. We used the CDR to validate the dementia diagnosis of the patients. CDR is a six-point scale used to assess six domains of cognitive and functional performance applicable to Alzheimer’s disease and related dementias [59–61]. Two Norwegian studies have shown that CDR staging is a valid substitute for a dementia assessment among nursing-home patients to rate dementia and dementia severity [60, 61]. The MMSE was used to assess global cognition and consists of 20 items concerning orientation, word registration and recall, attention, naming, reading, writing, following commands and figure copying. It can be scored between zero and 30, where a higher score indicates better performance [62]. The Neuropsychiatric Inventory questionnaire (NPI-Q) was used to assess the presence and severity of *behavioural and neuropsychiatric symptoms* common in dementia [63, 64]. The scale consists of 10 items. Each item represents a symptom and is rated as present or not (zero). If present, the severity is graded as mild (1) , moderate (2) or severe (3). Thus the minimum score is 0 (no symptoms at all) and the maximum score is 30. We also used sub- scales of the NPI-Q in the analyses, based on a previous large principal component analysis conducted with data from Norwegian nursing home patients [23]. The subscales were: 1) agitation: consisting of the items agitation/ aggression, irritability, and disinhibition (range: 0–9) 2) affective: consisting of the items depression and anxiety (range 0–6), and 3) apathy: The symptom apathy was analysed on its own.

### Depression and quality of life

Cornell Scale for Depression in Dementia [65] was used to assess *depression* in the participants. The scale contains 19 depressive symptoms and each item is rated on a scale from ‘absent’, ‘mild/ intermittent’ to ‘severe’. The minimum score is thus zero and maximum score is 38. According to a Norwegian nursing home study, a Cornell scale score of more than 7 points signifies depression [66]. “The quality of life in late-stage dementia scale”, QUALID [67] a proxy-rated scale was used to measure the QoL. The informants were professional caregivers who knew the patient well and had spent at least three of the last seven days with the patient. The scale consists of 11 items with a possible score of one to five on each item, which gives a total possible score range from 11 to 55. A lower score indicates a higher quality of life.

Demographic factors: participant age, gender, previous and present medical history, and the length of stay in a nursing home (from date of admission at the current nursing home).

### Ethics

The study was approved by the Regional Committee for Medical Ethics in south east of Norway September 2012. The nursing staff at the respective participating nursing home allocated eligible candidates, provided information about the study and gathered written consent. Primary caregiver provided written and verbal information about the study to the patients and their relatives. All the participants gave written consent to participate. Surrogate consent procedure was not used. The Regional Committee for Medical Ethics in south east of Norway approved this consent procedure.

### Data analysis

A power calculation was made using the BBS results of a pilot study from a nursing home in Norway. An analysis with 80 % power, alpha of 0.05, and an average difference of 2.5 points (SD = 4.2) between the intervention group and the control group, indicated that we should include 70 participants in each group to account for some degree of lost to follow-up. We did a complete responder analysis: all the participants who dropped out during the intervention and follow-up period were excluded (see flow chart Fig. 1). We assessed variables at baseline to establish whether the randomization procedure was successful.

The long-term effects of the intervention and detraining on each outcome were assessed with a linear mixed model for repeated measurements. The statistical model contained a random intercept, the outcome variable at baseline as a covariate and the fixed factors effects group affiliation, time and the interaction between time and group affiliation. We used the robust estimation of the standard error. The adjusted means at time 1 (immediately after intervention completion) and time 2 (3 months after intervention completion) and the mean differences within and between groups were estimated from the statistical model using estimated linear combinations. All statistical analyses were performed with the IBM SPSS Statistics version 20 or STATA version 14.

## Results

### Demographic and baseline scores

The background characteristics and baseline results of the assessments of the participants are reported in Table 1. The intervention and the control group were similar at baseline. The characteristics of the 16 participants who were not included in the ITT analyses were not significantly different from the final ITT population. The average age of the participants was 86.9 years (7.4) and almost three out of four were women. Thirty-one percent was able to walk independently without walking aid and less than 10 % used a wheel chair. The average score on Berg Balance Scale was 35 points points and two thirds scored less than 45 at baseline, which means they are at increased risk of falling [68]. Ninety-four percent of the participants walked slower than 0.8 m per second, which means an increased risk of frailty [69]. The men scored higher than the women at baseline on 30-s CST (6.8 rises vs. 5.8), however the difference was not statistically significant. See Table 1 and Telenius et al., 2015 [41] for more details regarding demographic information and baseline results.

**Table 1:** Baseline characteristics

	Whole sample *n* = 170	Intervention *n* = 87	Control *n* = 83	*p*-value
Age years mean (SD)	86.9 (7.4)	87.3 (7.0)	86.5 (7.7)	0.48
Female n (%)	125 (73.5)	63 (72.4)	62 (74.7)	0.74
NH stay months mean (SD)	25.7 (24.5)	23.8 (20.0)	27.6 (28.4)	0.34
Walk independently n (%)	52 (31.1)	25 (28.7)	27 (32.5)	0.70
No of diagnosis mean (SD)	3.4 (1.9)	3.4 (1.9)	3.3 (1.9)	0.78
No of medications mean (SD)	6.4 (3.4)	5.8 (3.1)	6.7 (3.6)	0.10
BBS points mean (SD)	34.8 (14.0)	34.3 (14.4)	35.3 (13.7)	0.65
CST points *n* = 161 mean (SD)	6.1 (3.0)	6.0 (3.1)	6.2 (2.9)	0.73
NWS m/ s mean (SD)	0.5 (0.2)	0.5 (0.2)	0.5 (0.2)	0.93
BI points mean (SD)	13.5 (3.5)	13.5 (3.5)	13.4 (3.6)	0.83
MMSE points mean (SD)	15.6 (4.9)	15.5 (0.6)	15.7 (4.9)	0.81
QUALID points mean (SD)	18.1 (5.8)	18.4 (6.1)	17.7 (5.4)	0.39
Cornell Scala points mean (SD)	4.9 (4.5)	4.9 (4.8)	4.9 (4.2)	0.98
NPI points mean (SD)	5.5 (5.4)	6.1 (6.1)	4.8 (4.6)	0.14
Affective, NPI subscale	1.0 (1.4)	1.2 (1.5)	0.8 (1.3)	0.13
Agitation, NPI subscale	1.5 (1.9)	1.7 (2.1)	1.3 (1.7)	0.16
Apathy, NPI subscale	0.5 (0.8)	0.6 (0.8)	0.4 (0.7)	0.08
Affective symptoms present n (%)	80 (47.1)	44 (50.6)	36 (46.4)	0.35
Agitation symptoms present n (%)	134 (78.8)	69 (79.3)	65 (78.3)	0.88
Apathy symptoms present n (%)	57 (33.5)	34 (39.1)	23 (37.7)	0.12

Independent samples t-test were used for continuous data and χ^2^ on categorical data

*BBS* Berg Balance Scale, *CST* Chair stand test, *NWS* Normal walking speed, *BI* Barthel Index, *MMSE* Mini Mental State Examination, *NPI* Neuropsychiatric Inventory

### Attendance

The persons in the exercise group participated on average in 18.1 sessions (Minimum 0- maximum 24, SD 6.8). This gives an attendance rate of 75 %. Severity of dementia, depression, functional balance or neurological disease did not influence the attendance rate. No adverse effects of exercise were observed. The control group participated on average in 16.4 sessions (minimum 4- maximum 24, SD 5.8). For more information about the attendance and intensity of exercise sessions, please see Telenius et al., 2015 [41].

### Lasting effects *within* the exercise and control group

Table 2 shows the adjusted means at time 1 (T1) and time 2 (T2) for both groups. When considering the within group changes in the EG, the results reveal a non-significant improvement in balance function by 2.7 points from baseline to 6 months follow-up. There was also an improvement in leg strength in the same period: The CST-score from baseline to 6 months assessment improved by 0.8 rises in average. The EG deteriorated on both BBS (1.5 points) and CRT (0.4 points) during the detraining period, however, these changes were also non-significant. When considering the mental health variables, the results in the EG demonstrate a non-significant improvement in NPI-Q-scores (including all its sub scores) from baseline to 6 months follow-up. There was a slight improvement during exercise cessation on the subscale of affective symptoms.

**Table 2:** Results from baseline (T0), 3 months (T1) and 6 months (T2) for exercise and control group

Variable	T1: 12 weeks	Exercise		Control		Difference between groups at T0, T1 and T2	*P*-value T2
	T2: 6 months						
		Means (SD)/ Adjusted means (CI)	Within group change T1T2	Means (SD)/ Adjusted means (CI)	Within group change T1T2		
Berg	T0	34.3 (14.4)	−1.5 (−3.1–0.2)	35.3 (13.7)	−2.2 * (−4.2--0.3)	1.0	
	T1	38.4 (36.8–40.1)		36.2 (34.7–37.6)		2.3	
	T2	37.0 (35.0–38.9)		33.9 (32.0–35.9)		3.0	0.031*
CST	T0	6.0 (3.1)	−0.4 (−0.9–0.3)	6.2 (2.9)	−0.3 (−1.1–0.3)	0.2	
	T1	7.1 (6.6–7.7)		6.5 (6.0–7.1)		0.6	
	T2	6.8 (6.2–7.4)		6.2 (5.4–6.9)		1.7	0.175
NWS	T0	0.5 (0.2)	−0.02 (−0.04–0.02)	0.5 (0.2)	−0.01 (−0.1–0.03)	0	
	T1	0.49 (0.46–0.51)		0.49 (0.44–0.54)		−0.001	
	T2	0.47 (0.44–0.51)		0.46 (0.43–0.5)		0.01	0.685
Barthel	T0	13.5 (3.5)	−0.6 (−1.3–0.1)	13.4 (3.6)	−0.8 (−1.6–0.03)	0.1	
	T1	13.7 (13.0–14.3)		12.9 (12.3–13.5)		0.8	
	T2	13.0 (12.3–13.8)		12.1 (11.2–12.9)		1.0	0.082
MMSE	T0	15.5 (0.6)	−1.0* (−1.9–0.2)	15.7 (4.9)	−1.4* (−2.4--0.5)	0.2	
	T1	15.4 (14.5–16.3)		15.3 (14.6–16.1)		0.1	
	T2	14.4 (13.5–15.2)		13.9 (12.9–14.9)		0.5	0.492
NPI sum	T0	6.1 (6.1)	0.03 (−1.3–1.3)	4.8 (4.6)	0.7 (−0.8–2.3)	1.3	
	T1	4.8 (3.7–5.8)		5.6 (4.1–7.1)		−0.9	
	T2	4.8 (3.8–5.8)		6.4 (5.1–7.7)		−1.6	0.059
Affective	T0	1.2 (1.5)	−0.04 (−03–02)	0.8 (1.3)	−0.1 (−0.3–0.4)	0.4	
	T1	0.9 (0.6–1.2)		1.0 (0.7–1.4)		−0.1	
	T2	0.9 (0.6–1.1)		1.1 (0.8–1.4)		−0.2	0.257
Agitation	T0	1.7 (2.1)	0.04 (−0.5–0.5)	1.3 (1.7)	0.2 (−0.3–0.8)	0.4	
	T1	1.3 (0.9–1.7)		1.8 (1.3–2.3)		−0.5	
	T2	1.4 (1.0–1.8)		2.0 (1.5–2.5)		−0.7	0.045*
Apathy	T0	0.6 (0.8)	0.08 (−0.03–0.3)	0.4 (0.7)	0.15 (−0.1–0.3)	0.2	
	T1	0.3 (0.2–0.4)		0.4 (0.2–0.6)		−0.1	
	T2	0.4 (0.3–0.6)		0.5 (0.3–0.7)		−0.1	0.688
QUALID	T0	18.4 (6.1)	−0.2 (−1.6–1.2)	17.7 (5.4)	1.1 (−0.2–2.6)	0.7	
	T1	18.1 (16.8–19.3)		17.7 (16.4–19.0)		0.4	
	T2	17.8 (16.7–19.0)		18.8 (17.3–20.2)		−0.9	0.326
Cornell Scala	T0	4.9 (4.8)	0.8 (−0.8–2.4)	4.9 (4.2)	1.4* (0.1–2.6)	0	
	T1	4.0 (2.6–5.4)		3.8 (2.8–4.7)		0.2	
	T2	4.8 (3.8–5.8)		5.1 (4.0–6.3)		−03	0.668

Results at T0 are given as mean (SD). Results at T1 and T2 are estimated means (95 % confidence interval) or mean difference (95 % confidence interval) derived from the linear mixed model

*BBS* Berg Balance Scale, *CST* Chair stand test, *NWS* Normal walking speed, *BI* Barthel Index, *MMSE* Mini Mental State Examination, *NPI* Neuropsychiatric Inventory Questionnaire

T0 = baseline, T1 = 12 weeks, T2 = 6 months

*p < 0.05

The CG reduced their BBS score in both periods: On average they dropped 1.4 points from baseline to 6- months follow-up and 2.2 points (*p* = 0.022) after intervention cessation. The CG deteriorated on ADL-function measured by BI: From baseline to 6 months follow-up the score reduced by 1.3 points and after intervention cessation, 0.8 points. NPI sum score and all sub-scores deteriorated from baseline to 6 months follow-up and the CG also declined significantly on the Cornell Scale (*p* = 0.034).

### Comparison of lasting effects *between* exercise and control group

From baseline to 6 months follow-up, the EG improved their BBS score while the CG declined and the difference between the groups was statistically significant (*p* = 0.031). While the CG remained unchanged on the CST during the 6 months period, the EG improved somewhat on CST, but the difference was not significant. After 6 months the EG scored significantly better than the CG on the NPI sub-score agitation (*p* = 0.045). The difference in NPI sum score was close to significant (*p* = 0.059).

## Discussion

### Long-term effect on physical function

This study demonstrates that a 12-week intensive functional exercise program had a long-term effect after three months of detraining on balance in a population of nursing home residents with dementia. Balance was the predetermined primary outcome. An effect of exercise on balance function was also found immediately after 12 weeks of exercise [41]. The change in BBS from baseline to 6 months follow-up was 2.7 points in the exercise group. To the authors knowledge, clinically meaningful change has not been determined for BBS, however minimal detectable change for dependent institutionalized elderly has been reported to be 7.7 points in a Swedish study [70]. This means that due to intra-person variability of balance function from one day to another, a change in BBS score should be at least 8 or higher in dependent nursing home residents to represent a true change. Even though the EG had a slight improvement in lower limb strength (CST) and the CG deteriorated during the 24 weeks, the difference in change between the groups was not significant and both groups declined during detraining [41]. The deterioration in strength after exercise cessation has been demonstrated in other studies [44, 71]. An RCT using the HIFE-programme in a population of 191 nursing home residents including 50 % patients with dementia demonstrated long-term effect on walking speed, balance (BBS) and lower limb strength (1RM) [50]. The exercise group improved on both balance and strength during the detraining period while the control group improved in balance and declined in strength [50]. In contrast to our study, the design of the study by Rosendahl et al., 2006 [50], included continuous encouragement of the participants to do tasks to maintain physical function for another three months after the intervention period. This could explain their positive long-term effects. In our study, the withdrawal of intervention might have led to a disappointment for some of the EG-participants, they may have lost interest and become inactive which led to the decline in balance and strength after exercise cessation. It is however interesting to notice that, even though both EG and CG deteriorated on almost all variables during detraining period, the CG decline was of greater extent, indicating that even terminated exercise has some effect. Preservation of effect from functional exercises has also been demonstrated in community-dwelling elderly women [43]. DeVreede et al., 2005 [43], found that the group who did functional exercises scored better than resistance-training group and inactive control group on Assessment of Daily Activity Performance (ADAP) six months after training ended. The ADAP provides a total score and five physical domain scores: upper body strength, lower body strength, flexibility, endurance, and balance and coordination. It is likely that the effect is easier to maintain due to the functional nature of these exercises which means that they are repeated throughout the day even when not exercising.

### Long-term effect on mental health

From baseline to 6 months assessment, the EG improved on the NPI sum score and all sub-scores while the CG deteriorated on these scores. The differences between groups were significant for the agitation sub-score. An effect of exercise on apathy was found at 12 weeks [41], but at 6 months there was no longer a statistically difference between groups. High quality RCTs are lacking, however smaller uncontrolled and observational studies suggest effect on agitation of walking in groups [72] and continuous program activities including physical exercise [73]. A pilot study with chair-exercises did not demonstrate any effect on agitation [74], but this might be due to too low intensity. A review of effect of exercise on behavioural and psychological symptoms in dementia states that exercise appear to be beneficial in reducing depressed mood, agitation and wandering, however the review emphasizes that the methodological shortcomings of work in this area are substantial [36].

The results from our study show that it is possible to influence on neuropsychiatric symptoms by other means than medication and this is important due to the overuse of antipsychotics to treat behavioural problems in persons with dementia [75, 76]. The modest effect of these drugs coupled with the increase in risk of adverse effects argues for a shift towards other treatment modalities [19, 77]. Encouragement and stimulation to be physically active is of great importance, and access to rehabilitation staff seems crucial to achieve improvements in function among nursing home residents [78]. Nursing home residents are a frail group that often has difficulty carrying out physical training on their own. In order to maintain positive results physical training should be an on-going activity.

### Limitations of the study

There are limitations in the present study. First of all our inclusion criteria restrict our findings to nursing home residents with the ability to rise from a chair with the maximum help from one person and being able to walk for 6 m with or without walking aid. This means that the frailest residents have not been included, even though some of the participants used electrical wheel chairs and managed to move six meters only with great help from support walkers.

The recruitment through meetings and direct invitations may also introduce some bias in generalizations. Each nursing home was asked to recruit up to 12 participants. This may have led to recruitment of only the healthiest. However only three out of 18 nursing homes managed to recruit 12 participants, so it is unlikely that this strategy has influenced the characteristics of the population. It is difficult to ensure homogeneity of the intervention since a total of 27 physiotherapists were involved in the execution of the intervention and 18 professional caregivers carried out the control activity.

The amount of formal tests is high in this study and, since this could lead to alpha inflation, the secondary endpoints should be interpreted as descriptive statistics and not formal probabilities. One way to adjust for the high number of variables is to use the Bonferroni correction [79]. According to Bonferroni the modified *p*-value at 95 % confidence when testing 11 variables is 0.0045. At this significance level the results from the current study are not statistically significant, however there are strong indications of effect of exercise on neuropsychiatric symptoms that should be investigated further as a primary outcome.

The frequency of training sessions and the long period of intervention (12 weeks) strengthened the study, but it may be questioned whether three months of intervention is a sufficient time period for optimal benefit. We did not gather information about the habitual physical activity of our participants, thus we cannot control for whether the level of physical activity in addition to the exercise contributed to the results. The physical outcome measures may have been affected by the reduced amount of motivation and/ or understanding in some participants. It is also a limitation of the study that some of the questionnaires may have been filled out by nursing staff with limited knowledge and experience with the different instruments. Assistance was offered, but it is not certain that all involved felt comfortable to contact project leader to attain wanted information.

### Strengths of the study

Strengths of the study are factors such as blinding of testers, randomization and analysis by intention to treat. The linear mixed model for repeated measurements is effective in accommodating missing data [80]. However, a complete case analysis demonstrated the same results as presented, and this indicates that the effect of missing was minimal. In each group, data from T1 or T2, or both, were missing for 15 participants. The participants with missing data were slightly older, scored higher on MMSE and lower on Cornell scale, but the differences were not statistically significant. The high attendance and low dropout rate is a strength of the study, and so is the use of simple and inexpensive equipment. In addition, by employing local physiotherapists, we have demonstrated that effect can be achieved at most nursing homes with access to basic gym equipment and physiotherapy recourses.

## Conclusion

The results indicate long-time positive effects of a high intensity functional exercise program on balance. A possible effect of exercise on agitation is demonstrated after an intervention period of 12 weeks followed by a detraining period of 12 weeks. Future research should focus on exploring the effect of exercise on mental health in the population of nursing home residents with mild and moderate dementia.
